# Utility of routine data sources for feedback on the quality of cancer care: an assessment based on clinical practice guidelines

**DOI:** 10.1186/1472-6963-9-84

**Published:** 2009-05-27

**Authors:** Michael Coory, Bridie Thompson, Peter Baade, Lin Fritschi

**Affiliations:** 1 School of Population Health, The University of Queensland, Queensland, Australia; 2 Statistical Analysis Unit, Queensland Health, Queensland, Australia; 3 Viertel Centre for Research in Cancer Control, Cancer Council, Queensland, Australia; 4 Western Australian Institute for Medical Research, The University of Western Australia, Perth, Australia

## Abstract

**Background:**

Not all cancer patients receive state-of-the-art care and providing regular feedback to clinicians might reduce this problem. The purpose of this study was to assess the utility of various data sources in providing feedback on the quality of cancer care.

**Methods:**

Published clinical practice guidelines were used to obtain a list of processes-of-care of interest to clinicians. These were assigned to one of four data categories according to their availability and the marginal cost of using them for feedback.

**Results:**

Only 8 (3%) of 243 processes-of-care could be measured using population-based registry or administrative inpatient data (lowest cost). A further 119 (49%) could be measured using a core clinical registry, which contains information on important prognostic factors (e.g., clinical stage, physiological reserve, hormone-receptor status). Another 88 (36%) required an expanded clinical registry or medical record review; mainly because they concerned long-term management of disease progression (recurrences and metastases) and 28 (11.5%) required patient interview or audio-taping of consultations because they involved information sharing between clinician and patient.

**Conclusion:**

The advantages of population-based cancer registries and administrative inpatient data are wide coverage and low cost. The disadvantage is that they currently contain information on only a few processes-of-care. In most jurisdictions, clinical cancer registries, which can be used to report on many more processes-of-care, do not cover smaller hospitals. If we are to provide feedback about all patients, not just those in larger academic hospitals with the most developed data systems, then we need to develop sustainable population-based data systems that capture information on prognostic factors at the time of initial diagnosis and information on management of disease progression.

## Background

There is a perception, supported by some evidence, that not all cancer patients receive state-of-the-art care.[1-3] Providing regular feedback to clinicians might reduce this problem[1] and supporting this contention is a Cochrane review, which found that regular feedback can provide moderate, but important improvements in quality-of-care.[4] Recent studies from the United States also suggest that timely reporting and feedback improves quality-of-care.[5,6]

Feedback about quality of care needs to be done at a sustainable cost, which is not an easy task. For example, in 1990, California's assembly debated new requirements for reporting clinical indicators to assess quality of care. When the cost of collecting the data items for the clinical indicators was estimated at $61 M, fiscal reality intervened and the legislature mandated the use of quality measures that used California's existing routinely-maintained databases.[7]

The aim of this current study was to determine what data are needed to provide feedback on measures of interest to clinicians, who treat cancer patients. Feedback cannot be done without data; hence it is important to understand the utility of current data sources. We grouped data sources into four categories based on their availability and the marginal cost of using them for feedback. Published clinical practice guidelines were used to identify processes-of-care of interest to clinicians.

We were particularly interested in the utility of population-based cancer registries and administrative inpatient data, which are attractive sources of data for feedback because they have wide coverage. Also, because they are maintained primarily for other purposes, they can provide feedback at small marginal cost.

Several studies have assessed the quality of data in various databases designed to measure quality of cancer care.[8-10] To our knowledge, this is the first study to describe the utility of various data sources in providing feedback on cancer care.

## Methods

### Categories of data

Possible sources of data for regular feedback include administrative inpatient databases, population-based cancer registries, (hospital-based) clinical cancer registries, medical record review, patient interview and audio-taping of consultations. We grouped these data sources into four categories based on the marginal cost of using them for feedback from least (*Category 1*) to most expensive. (Table 1)

**Table 1:** Categories of data to measure adherence to guidelines

	Data items
	
*Category 1*	
Population-based cancer registry, with or with linkage to administrative inpatient data	Demographics
	Site of cancer
	Morphology
	Summary staging information at time of diagnosis (local, regional, distant spread)
	Type and date of inpatient surgery
	Co-morbidities (pre-existing and complications)
*Category 2*	
Core clinical registry	Type and date of initial treatments
	Intent of treatment (curative versus palliative)
	Clinical (TNM) stage at diagnosis
	Performance status
	Specific prognostic factors (e.g., hormone receptor status for breast cancer)
	Assessment by multidisciplinary team
	Participation in clinical trial

*Category 3*	
Extended clinical registry or medical record review	Long-term follow up
	Management of disease progression
	Management of complications of cancer therapies (e.g., anti-emetics, heparin)
	Details of surgical techniques (e.g., omental covering of anastomosis, on-table colonic lavage)

*Category 4*	
Patient interview	Information sharing between clinicians and patients, for example
Audio-taping of consultation	• Method of breaking bad news
	• Inquiries about how spouse and children were coping
	• Resolution of family conflicts
	• Information provided to help woman choose between breast-conserving surgery and mastectomy

The primary purpose of administrative inpatient data is related to billing patients and funding hospitals[7], while for population-based cancer registries (at least in most jurisdictions to date) the primary purpose is to measure incidence, prevalence, mortality and population-based relative survival.[11] Therefore they are secondary data sources for the purpose of measuring quality of cancer care, and can therefore provide this information at low marginal cost. In contrast, the primary purpose of the other data sources, listed in Table 1, is to measure the quality of cancer care and consequently, there are no cost offsets. We ranked a "core clinical registry" as less costly than an "extended clinical registry" because the data items that define an "extended clinical registry" are more difficult and time-consuming to collect and therefore more costly.

As with any categorisation scheme, it is a useful framework, but we make no claim that it is perfect. For example, the distinction between population-based and (hospital-based) clinical cancer registries is less clear-cut than it used to be[12] with some population-based registries augmenting their data with information on clinical stage.[8] However, because this is not widespread in most jurisdictions (at least at present) we assigned feedback measures reliant on clinical stage to *Category 2*: core clinical registry; whereas those based on broader staging information (e.g., regional or distant spread) such as recorded by SEER[13] were assigned to *Category 1*.

Clinical registries are also constantly evolving and can vary. To identify data items that could reasonably be assumed to comprise a typical clinical registry, we used the Minimum Data Set for Clinical Cancer Registration from New South Wales, Australia.[14] It is based on clinical registries in the United States, United Kingdom and Canada and it represents a fairly typical minimum clinical data set. Under our schema, The National Cancer Data Base (American College of Surgeons) would be classified as a core clinical registry. It collects information on patient characteristics, tumour staging and histology, type of first course treatment administered, disease recurrence and survival.[15]

Clinical registries have emphasised *initial *diagnostic procedures and *initial *therapies because they were thought to have the greatest influence on survival.[16] We therefore allocated processes-of-care related to longer term follow-up or management of recurrences or metastases to *Category 3*: extended clinical registry or medical record review (Table 1). Also included in *Category 3*were guidelines based on surgical techniques, because unlike the date and type of inpatient surgery, they are not captured in routine-inpatient data (i.e., *Category 1*).

Finally, we assigned to *Category 4 *all those guidelines that dealt with information sharing between clinicians and patients. These were mainly guidelines recommending that the clinician communicate certain information to the patient so that he or she could make an informed decision. For example, one of the melanoma guidelines is that patients should be provided with adequate information about prognosis because this is associated with enhanced patient well-being. While such information could be routinely collected if clinicians documented their discussions and recommendations in a standard way, this is currently the exception, rather than standard practice and so typically requires costly, one-off studies. [17]

### Processes-of-care

To obtain a list of processes-of-care of interest to clinicians, we used published Australian guidelines for the four of the five most common cancers diagnosed in Australia; that is, colorectal[18], breast (published separately for early and advanced)[19,20], and lung cancer[21], and melanoma.[22] The Australian guidelines for prostate cancer (the commonest cancer among Australian men) are rudimentary in that they comprise only four guidelines, each of which relates to providing information to patients about treatment options for localised disease.[23] We therefore decided not to include prostate cancer guidelines in this study.

Where the Australian guidelines were evidence-based, they relied primarily on international studies, so their content should reflect processes-of-care of interest to clinicians in most jurisdictions. Where it was not possible for them to be evidence-based (i.e., evidence was lacking) they represent the consensus view of opinion leaders in the field and again should encompass aspects of cancer management of interest to clinicians in most jurisdictions. Although the quality of guidelines can vary across jurisdictions, there is much less scope for the content of the guidelines to vary. [24]

We were interested in the direct clinical management of cancer patients and therefore excluded guidelines that summarised information on risk factors, epidemiology, and the mechanics of various pathology procedures or investigation of patients with suspicious symptoms not yet proven to be cancer. In all, there were 315 guidelines, of which we excluded 72 (23%), leaving 243 guidelines to assess.

### Assessment

All four of us independently assigned each process-of-care (based on a guideline) to one of the four data categories. Given the clear-cut categorisation of data sources and data items (Table 1), disagreements were uncommon (<2% of feedback measures) and were discussed until consensus was reached. A process-of-care that could be assigned to more than one data category was assigned to the least costly category.

## Results

### Type of data required for measurement

Only 8 of 243 processes-of-care (3.3%) could be reported using a population-based registry or administrative inpatient data, either linked or unlinked (*Category 1*); Table 2. Although nearly half of the guidelines (119 or 49.0%) could be measured using a core clinical registry (*Category 2*), 88 (36.1%) required an expanded clinical registry or medical record review (*Category 3*) because they were based on information about follow-up or management of disease progression. A further 28 (11.5%) of the guidelines were classified to *Category 4*(patient interview or audio-taping of consultation).

**Table 2:** Data category by feedback measure and type of cancer

Data category	Type of guideline	n (%)					
		
		Early breast	Advanced breast	Colorectal	Lung	Melanoma	Total
*Category 1*	Level of hospital	0 (0.0%)	0 (0.0%)	0 (0.0%)	1 (1.2%)	0 (0.0%)	1 (0.4%)
Population-based registry, routine-inpatient data	Surgery; guideline not reliant on clinical stage	0 (0.0%)	0 (0.0%)	0 (0.0%)	4 (4.8%)	3 (7.3%)	7 (2.9%)
	***Sub-total***	***0 (0.0%)***	***0 (0.0%)***	***0 (0.0%)***	***5 (6.0%)***	***3 (7.3%)***	***8 (3.3%)***

*Category 2*	Initial diagnostic or staging procedures	0 (0.0%)	0 (0.0%)	2 (4.3%)	14 (16.9%	3 (7.3%)	19 (7.8%)
Core clinical registry	Initial treatment in relation to clinical stage-at-diagnosis or performance status or specific prognostic features of the cancer	15 (60.0%)	10 (21.3%)	23 48.9%))	23 (27.7%))	25 (61.0%)	96 (39.5%)
	Participation in clinical trial	0 (0.0%)	0 (0.0%)	0 (0.0%)	2 (1.2%)	0 (0.0%)	2 (0.8%)
	Assessment by multi-disciplinary team;	0 (0.0%)	0 (0.0%)	0 (0.0%)	2 (1.2%)	0 (0.0%)	2 (0.8%)
	***Sub-total***	***15 (60.0%)***	***10 (21.2%)***	***25 (53.2%)***	***41 (49.4%)***	***28 (68.3%)***	***119 (49.0%)***

*Category 3*	Details of surgery;	0 (0.0%)	0 (0.0%)	10 (21.3%)	0 (0.0%)	0 (0.0%)	10 (4.1%)
Extended clinical registry medical record review,	Treatments for complications of cancer treatment	0 (0.0%)	3 (6.4%)	2 (4.3%)	1 (1.2%)	0 (0.0%)	6 (2.5%)
	Long-term follow-up;	1 (4.0%)	3 (6.4%)	3 (6.4%)	2 (2.4%)	4 (9.8%)	13 (5.3%)
	Management of disease progression including pain relief and palliation	2 (8.0%)	22 (46.8%)	6 (12.8%)	29 (34.9%)	0 (0.0%)	59 (24.3%)
	***Sub-total***	***3 (12.0%)***	***28 (59.6%)***	***21 (44.9%)***	***32 (38.6%)***	***4 (9.8%)***	***88 (36.1%)***

*Category 4*	Communication and information sharing between clinician and patient	7 (28.0%)	9 (19.1%)	1 (2.1%)	5 (6.0%)	6 (14.6%)	28 (11.5%)
Patient interview, audio-taping of consultation							
	***Grand total***	***25 (100%)***	***47 (100%)***	***47 (100%)***	***83 (100%)***	***41 (100%)***	***243 (100%)***

The main reasons why population-based cancer registries and administrative inpatient data can be used to measure so few processes-of-care are shown in column 2 (*Type of guideline*) of Table 2. Numerically, two reasons stand out from the others. The first is the lack of information on prognostic factors such as clinical stage at diagnosis, physiological reserve (i.e., measures of performance status such as Karnofsky score or ECOG status) and hormone-receptor status. Specifically, 39.5% of the processes-of-care are reliant on reporting initial treatments in relation to these factors. The second is the lack of information about the management of disease progression (24.3%). Other reasons included the lack of information on initial diagnostic and staging procedures (7.8%) and long-term follow-up (5.3%).

There was variation across different cancers (Table 2). For example, only 9.8% of the melanoma guidelines required an extended clinical registry or medical record review (*Category 3*) because melanoma is less likely to progress than other cancers and hence there are fewer guidelines about management of progression. In contrast, 59.6% of the guidelines for advanced breast cancer were classified to *Category 3*, as were 44.9% of the guidelines for colorectal and 38.6% for lung.

## Discussion

Population-based registries and administrative inpatient databases are maintained primarily for other purposes and can therefore be used to provide feedback measures at small marginal cost. This is in contrast to clinical registries, for which feedback and audit are primary functions. One-off studies based on medical record review (which we classified to *Category 3*) are probably less sustainable as a means of providing regular feedback and the measures are likely to be less standardised and comparable across jurisdictions.

The results of this study show that there are two broad pieces of information that would allow feedback on more measures using population-based registries and administrative inpatient data: prognostic factors as measured at the time of diagnosis (e.g., clinical stage, performance status, hormone-receptor status); and information on treatments for disease progression (e.g., recurrences and metastases), not just initial treatments.

An important prognostic factor is clinical stage and addition of this data item to population-based registries is probably an achievable aim, for at least some types of cancers [25,26]. For example, a recent Australian report concluded that several types of cancers can be assigned a clinical TNM stage, based on the data already available to a population-based registry.[26]

Many clinical registries only collect data on initial treatments, but many guidelines concern the management of recurrences or metastases. Management decisions about recurrences or metastases can have an important effect on the quality-of-life of cancer patients and their carers [27], so that feedback about disease progression is arguably as important as feedback about initial treatment.

In recent years, more components of cancer care have moved from inpatient to ambulatory-care settings, making it more difficult to obtain complete and accurate data on cancer care[9]. For the purposes of this study we assumed that a core clinical registry could collect complete information on all initial treatments, even those done in ambulatory care settings. In practice, this might not be the case, with several studies showing that ambulatory care data are often incomplete, even in those clinical registries that make special attempts to collect such data [9,10,28,29].

We also assumed that population-based cancer registries and administrative inpatient data cover the entire population, as is the case in Australia. This is not the case in some countries like the United States where population-based cancer registries (SEER) are a representative sample and claims databases for inpatients typically cover only a segment of the population (e.g., Medicare claims data only includes patients 65 years or older).

We used clinical practice guidelines to identify processes-of-care of interest to clinicians. These would need to be developed into feedback measures, which involves expert clinical advice and clear exclusion criteria. This current paper has only assessed the utility of various data sources for feedback, which is only a small, albeit essential, part of the process.[30,31]

The starting point for this study was that sometimes there is a gap between what is currently provided and what might be provided in ideal circumstances to cancer patients. If we accept that timely and on-going feedback can reduce the gap, then we need systems that can routinely provide data that are of sufficient quality and cover all patients in a defined population, regardless of whether they are treated in small hospitals or in large hospitals with well-developed data systems.[9]

Systematic reviews have shown that for cancer, as for other conditions, patients tend to have poorer outcomes if they are treated by a hospital or clinician who treats a relatively small number of patients with a particular cancer.[32] Therefore, it is in the smaller hospitals where there are a-priori concerns about quality of cancer care, but it is usually only population-based registries or routine-inpatient databases that cover these hospitals.[11,33] Whether it is feasible to maintain clinical registries in smaller hospitals is debatable. This makes providing feedback measures to smaller hospitals problematic, although these might be the hospitals where the most gains in quality-of-care can be made.

## Conclusion

The advantages of population-based cancer registries and administrative inpatient data are wide coverage and low cost. The disadvantage is that they can currently be used to report only a few feedback measures. In most jurisdictions, clinical cancer registries, which can be used to report many more feedback measures, do not cover smaller hospitals. If we are to provide feedback about all patients, not just those in larger academic hospitals with the most developed data systems, then we need to develop sustainable population-based data systems that capture information on prognostic factors at the time of initial diagnosis and information on management of disease progression.

## Competing interests

The authors declare that they have no competing interests.

## Authors' contributions

All authors participated in the design of the study. BT collated the data and MC conducted the analysis. MC prepared the initial draft. All authors contributed to critically revising the manuscript and read and approved the final manuscript.

## Pre-publication history

The pre-publication history for this paper can be accessed here:


